# Complete mitochondrial genome and phylogenetic position of the Levanidov’s charr *Salvelinus levanidovi* Chereshnev, Skopetz et Gudkov, 1989 (Salmoniformes, Salmonidae)

**DOI:** 10.1080/23802359.2020.1780979

**Published:** 2020-06-17

**Authors:** Alla G. Oleinik, Lubov A. Skurikhina, Andrey D. Kukhlevsky, Alexander A. Semenchenko

**Affiliations:** a A.V. Zhirmunsky National Scientific Center of Marine Biology, Far Eastern Branch, Russian Academy of Sciences, Vladivostok, Russia; b Far Eastern Federal University, Vladivostok, Russia

**Keywords:** charr genus *Salvelinus*, Levanidov’s charr, *Salvelinus levanidovi*, mtDNA, phylogeny

## Abstract

The complete mitochondrial genome was sequenced in Levanidov’s charr *Salvelinus levanidovi*. The genome sequences are 16,624 bp, and the gene arrangement, composition, and size are similar to the charr genomes. The level of divergence between *S. levanidovi* and charr belonging to the genus *Salvelinus* was in the range from 4.80% to 3.65%. Molecular phylogeny provides new evidence that *S. levanidovi* is closely related to the common ancestor of the genus *Salvelinus*. The present study confirms that *S. fontinalis, S. levanidovi, S. leucomaensis*, and *S. namaycus* form a basal group of taxa, each of them belongs to an independent evolutionary line.

The Levanidov’s charr *Salvelinus levanidovi* was first described as an endemic species inhabiting a small range in rivers of the northern basin of the Sea of Okhotsk (Chereshnev et al. 1989). According to comparative data, *S. levanidovi* is characterized by the prevalence of plesiomorphic morphological characters (Chereshnev et al. 2002). In addition, *S. levanidovi* has preserved a primitive karyotype, which is also typical of *S. namaycush* and *S. fontinalis* (Frolov and Frolova 2004), and the ancestral mitochondrial genome (Oleinik et al. 2003). Molecular phylogenies, inferred from different DNA markers using various methods of analyses, indicate that basal position within the genus could be occupied by any of the following four species: *S. fontinalis, S. levanidovi, S. leucomaensis,* or *S. namaycus* (e.g. Oleinik, Skurikhina, et al. 2015; Osinov, Senchukova, et al. 2015, and references therein). Therefore, to research higher-level relationships among *Salvelinus* and to find out which of the charr species is the ‘most basal clade’, it is important to obtain the complete mitochondrial genome of *S. levanidovi.*

We sequenced and described the complete mitogenome of *S. levanidovi* for the first time in this study. *Salvelinus levanidovi* were collected in the middle flow of the Yama River (Sea of Okhotsk basin, Russia; 59°41′N, 154°21′E), a type locality of Levanidov’s charr (Chereshnev et al. 1989). The fish specimen is stored in the collection of the Genetics Laboratory, NSCMB FEB RAS, Vladivostok, Russia (www.imb.dvo.ru) with accession number LVYA94.030. Totally 5 pairs of primers were used, which were designed based on public sequences available in the GenBank for salmonid fish. The sequenced fragments were assembled into a complete mitogenome and annotated by comparing with published mitogenomes of charr using Geneious R11 (http://www.geneious.com/).

The complete mitogenome of native *S. levanidovi* was 16,624 bp in length. Like charr mitogenomes (Balakirev et al. 2016; Oleinik et al. 2019), the overall base composition was 28.1% of A, 26.4% of T, 28.6% of C, and 16.9% of G with a slight A + T bias (54.5%). The comparison of complete mitogenome was obtained with 20 mitogenomes of related groups available in the GenBank, including genera *Salvelinus*, *Parahucho*, and *Salmo* point to the relationships of *S. levanidovi* to charr species (Figure 1). *Salvelinus levanidovi* was phylogenetically positioned with other charr, showing a clear divergence from them. The average level of total sequence divergence (*D_xy_*) between them was 0.0390 ± 0.001; these values correspond to the level of interspecific variability in the genus (Oleinik, Skurikhina, et al. 2015; Osinov, Senchukova, et al. 2015). The highest divergence was found between the mitogenomes of *S. levanidovi* and *S. fontinalis* (0.0480 ± 0.0016) as well of *S. levanidovi* and *S. leucomaenis* (0.0423 ± 0.0016). At the same time, our *S. levanidovi* specimen showed similar sequence divergence (0.0365 ± 0.0015 on average) from other charrs in GenBank, including charrs from East Asia to North American. Вased on the variability of mitogenomes, *S. fontinalis, S. levanidovi, S. leucomaensis,* and *S. namaycus* form a basal group of taxa, each of them belonging to an independent evolutionary line. The present study confirms that *S. levanidovi* is closely related to the common ancestor of the genus *Salvelinus*.

**Figure 1.**
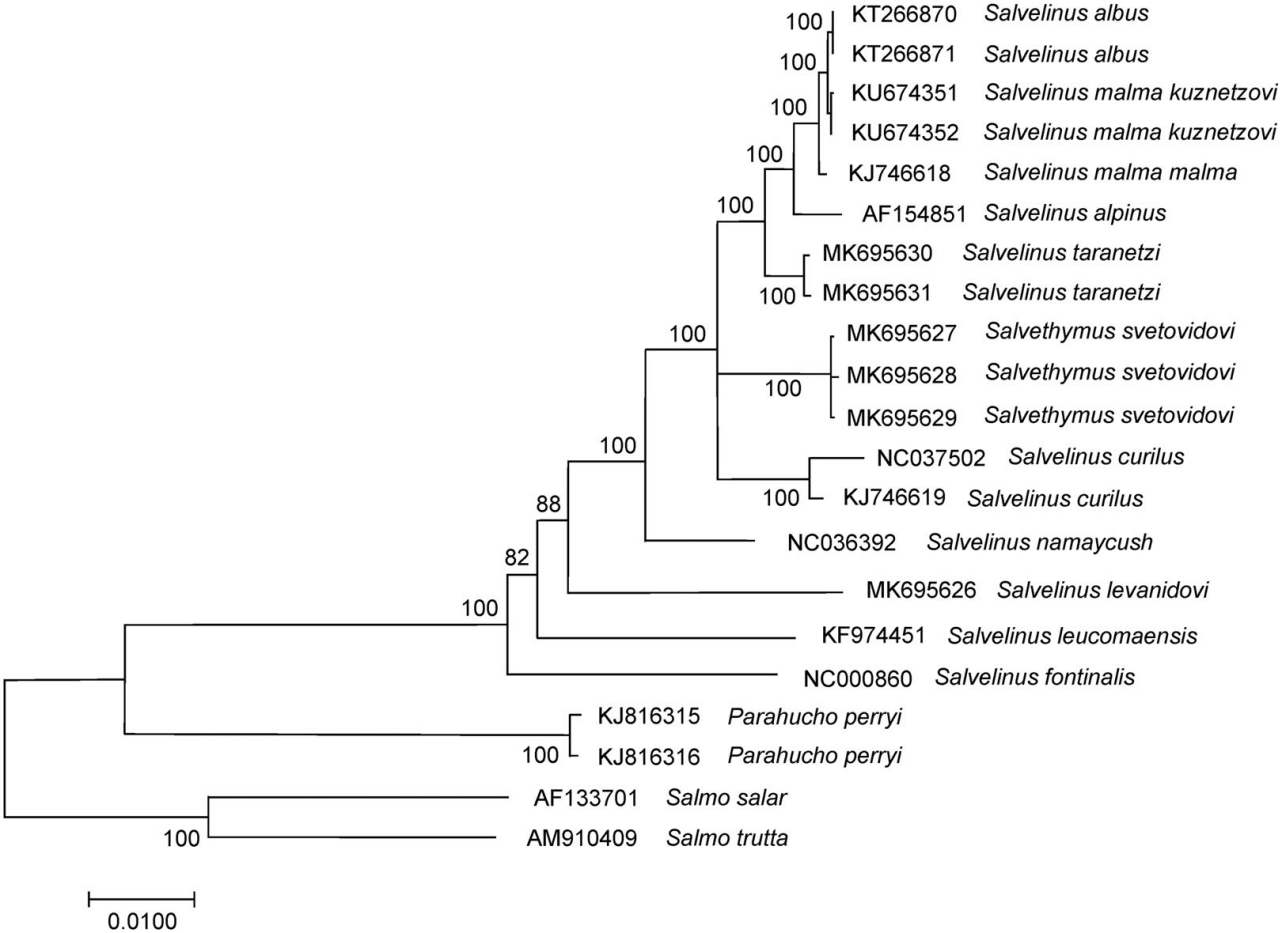
Maximum likelihood (ML) tree constructed based on the comparison of complete mitochondrial genome sequences of *S. levanidovi* and other GenBank representatives of the family Salmonidae. The tree is based on the GTR plus gamma plus invariant sites (GTR + G + I) model of nucleotide substitution. Genbank accession numbers for all sequences are listed in the figure. Numbers at the nodes indicate bootstrap probabilities from 1000 replications (values below 80% are omitted).

## Data Availability

The data that support the findings of this study are openly available in the National Center for Biotechnology Information database (NCBI/GenBank) at https://https.ncbi.nlm.nih.gov/, reference number MK695626.
